# Transradial and Transfemoral Approach in Patients with Prior Coronary Artery Bypass Grafting

**DOI:** 10.3390/jcm9030764

**Published:** 2020-03-12

**Authors:** Rafał Januszek, Zbigniew Siudak, Krzysztof Piotr Malinowski, Wojciech Wańha, Wojciech Wojakowski, Mariusz Gąsior, Stanisław Bartuś, Dariusz Dudek

**Affiliations:** 1 University of Physical Education, Department of Clinical Rehabilitation, 31-571 Kraków, Poland; 2 2nd Department of Cardiology and Cardiovascular Interventions, University Hospital, 31-501 Kraków, Poland; stanislaw.bartus@uj.edu.pl (S.B.); mcdudek@cyfronet.pl (D.D.); 3 Faculty of Medicine and Health Sciences, Jan Kochanowski University, 25-516 Kielce, Poland; zbigniew.siudak@gmail.com; 4 Institute of Public Health, Faculty of Health Sciences, Jagiellonian University Medical College, 31-531 Kraków, Poland; krzysztof.piotr.malinowski@gmail.com; 5 Department of Cardiology and Structural Heart Diseases, Medical University of Silesia, 40-635 Katowice, Poland; wojciech.wanha@gmail.com (W.W.); wwojakowski@sum.edu.pl (W.W.); 6 3rd Department of Cardiology, Silesian Centre for Heart Diseases in Zabrze, School of Medicine with the Division of Dentistry in Zabrze, Medical University of Silesia in Katowice, 41-800 Zabrze, Poland; m.gasior@op.pl; 7 2nd Department of Cardiology, Jagiellonian University Medical College, 31-501 Kraków, Poland; 8 Department of Interventional Cardiology, Jagiellonian University Medical College, 31-202 Kraków, Poland

**Keywords:** femoral vascular access, PCI, prior CABG, procedure-related complications, radial vascular access

## Abstract

The relationship between periprocedural complications and the type of vascular access in patients with prior history of coronary artery bypass grafting (CABG) and treated with percutaneous coronary interventions (PCIs) is less investigated than in the overall group of patients treated with PCI. The aim of the current study was to assess the relationship between the type of vascular access and selected periprocedural complications in a group of patients with prior history of CABG and treated with PCIs. Based on a Polish nationwide registry of interventional cardiology procedures called ORPKI, the authors analyzed 536,826 patients treated with PCI between 2014 and 2018. The authors extracted 32,225 cases with prior history of CABG. Then, patients with femoral and radial access as well as right and left radial access were compared. This comparison was proceeded by propensity score matching (PSM). After PSM, a multifactorial analysis revealed that patients treated with PCI from femoral access were significantly more often related to periprocedural deaths (odds ratio [OR]: 1.79; 95%, confidence interval [CI]: 1.1–3.0, *p* = 0.02) and cardiac arrests (OR: 1.98; 95%, CI: 1.38–2.87, *p* < 0.001). After inclusion of the Killip class grade and the occurrence of cardiac arrests before PCI into the PSM, the significance remained for procedural related cardiac arrests (OR: 1.55; 95%, CI: 1.07–2.28, *p* = 0.022]). However, a comparison of right and left radial access showed no significant differences between procedure-related complications. It has been confirmed that there is a statistical association between femoral access (compared to radial access) and a higher rate of periprocedural cardiac arrests in patients with prior history of CABG treated with PCI.

## 1. Introduction

In several studies, it has been demonstrated that, compared to the transfemoral approach (TFA), cardiac catheterization and percutaneous coronary intervention (PCI) using the transradial approach (TRA) are associated with lower rates of vascular and bleeding complications, reduced mortality, earlier ambulation, improved patient satisfaction, cost benefits, shorter fluoroscopic time and lower contrast dose [1,2,3]. In selected analyses, which included hemodynamically unstable high-risk patients, significant differences between patients with TFA and TRA were not confirmed in clinical outcomes during the 5-year follow-up, nor were there cases of major bleeding [4]. Data on the role of TRA catheterization among patients who have previously undergone coronary artery bypass grafting (CABG) surgery are limited [2,5,6,7,8]. Although it has been suggested in observational studies that TRA PCI in patients who have previously undergone CABG surgery is feasible and safe, it is technically more challenging than procedures performed via TFA access [5,6,7,8]. It has been established over the last decades that coronary angiography and possible PCI procedures in patients following prior CABG operations were preferably performed with the use of TFA [9]. This substantially distinguishes the mentioned subgroup of patients from the overall group treated with PCI, for which TRA has become more popular in recent years [10]. Another issue typical for the group of patients with prior history of CABG is the fact that among patients with TRA, left TRA is more common than right. This difference within the overall group of patients treated with PCI, due to the most common arterial bypass, originates most commonly in the left subclavian artery. In recent years, the percentage of patients with prior history of CABG treated from TRA is still increasing. However, this growth is much slower than that observed in the overall group of patients treated with PCI [9]. Analyses concerning vascular access including a large group of patients with prior history of CABG are very limited. 

Therefore, in the current study, the authors aimed to assess the relationship between the type of vascular access and selected procedure-related complications. 

## 2. Methods

### 2.1. Study Design and Patient Population

**Figure 1:**
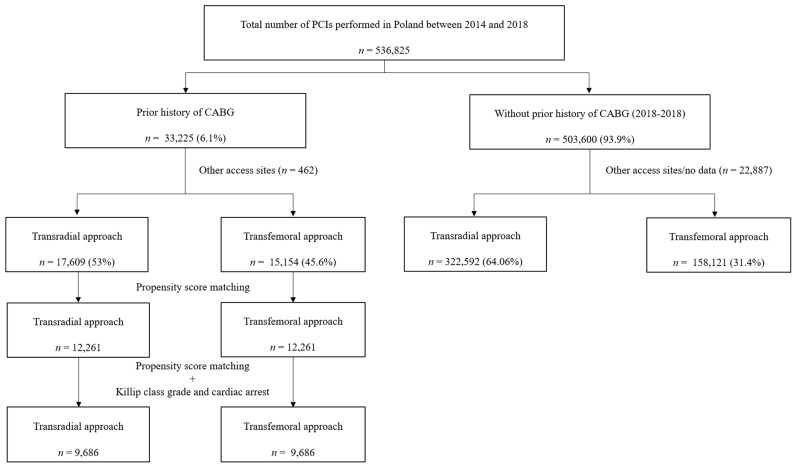
Flow of patients through the following stage of analysis. CABG = coronary artery bypass grafting; PCI = percutaneous coronary intervention.

This retrospective analysis was performed on prospectively collected data [10]. Data for conducting the current analysis were obtained from the national registry of percutaneous coronary interventions (ORPKI) maintained in cooperation with the Association of Cardiovascular Interventions (AISN) of the Polish Cardiac Society. The registry has been characterized in previously published papers [10]. The study covered data obtained from the registry between January 2014 and December 2018. The authors selected 32,225 patients with prior history of CABG out of 536,826 patients treated with PCI during the analyzed period. The patients’ flow in the current analysis is presented in Figure 1. The current study comprised all patients with previously implanted coronary bypass grafts, including arterial grafts, venous grafts or both. Moreover, patients without patent bypass grafts were included in the analysis. The technical aspects of the procedure, such as the choice of access site, sheath size and catheter side, were at the operator’s discretion. Furthermore, the periprocedural anticoagulation and indications for PCI, as well as stent type or use of distal protection devices, remained at the first operator’s discretion. The protocol complied with the Declaration of Helsinki, and all participants provided their written informed consent for participation in the trial prior to enrollment. Due to the fact that the data in the study were collected retrospectively, the consent of the Bioethics Committee was not required.

### 2.2. Study Endpoints

The main study endpoint was the assessment of the frequency of periprocedural mortality and other procedure-related complications in the group of patients with prior history of CABG and treated with PCI via vascular access. The authors compared TFA and TRA, as well as right and left vascular access. The secondary study endpoints included other indices related to vascular access, i.e., contrast dose and radiation exposure. 

### 2.3. Statistical Analysis

The groups were compared using the *t*-test for continuous variables or the *χ*^2^ test for nominal variables of their nonparametric equivalences when appropriate. Standardized differences were calculated for all baseline variables before and after matching. From all of the baseline/demographic characteristics, those with a *p* value lower than 0.2 for differences across groups were included in the logistic regression model used in propensity score matching (PSM). A logistic regression model was used to estimate the propensity scores. Scores are defined as the probability of using the femoral access site for each analyzed patient conditional on the baseline covariates. Then, for each patient with femoral access, one patient with radial access was assigned as a matched control. The assignment of these control patients was performed using the nearest neighbor algorithm (parameter r was set as 1 to match only one control (radial) to each with femoral access). One control (radial) patient was used only once, i.e., can be assigned to only one patient with femoral access. However, some patients with radial access could be discarded if they did not match any of the patients with femoral access. The groups were considered balanced if the standardized differences for each of the analyzed baseline/demographic characteristics were lower than 10%.

#### Femoral vs. Radial Access and Right vs. Left Radial Access

The effects of TFA on death, cardiac arrest, coronary artery perforation, puncture site bleeding and no reflow were assessed using mixed-effect models to account for matching. A similar analysis was performed for the right radial access. As a primary analysis, simple models with access site as the only fixed effect were constructed (model type A). Then, for the sensitivity analysis, additional models with procedure data used as covariates in the case of potential were associated with both the access site and the analyzed endpoints (*p* value < 0.2; model type B). Additionally, full models were constructed with all the procedure data as covariates and also any baseline/demographic characteristics with potential confounders (*p* value < 0.2; model type C).

## 3. Results

### 3.1. Population

Data for 15,154 (46%) procedures with TFA and 17,609 (54%) with TRA were extracted from the overall group of 32,225 patients treated with PCI and with prior history of CABG. Out of 32,225 patients following CABG, PCI was preceded by coronary angiography and bypassography in 28,217 patients (86.1%). Prior to PSM, it was noted that significantly older age (*p* = 0.002), more frequent incidence of diabetes (*p* < 0.001) and smoking history (*p* = 0.004) were among the characteristics for patients from the TRA group compared to TFA (Table 1). The rate of ST-segment elevation myocardial infarction (STEMI) patients before PCI was significantly higher in the TFA group in comparison to the TRA group (*p* < 0.001), while the rate of stable patients was significantly higher in the TRA group (*p* < 0.001; Table 1). Prior to PSM, the frequency of patients with Killip class IV was significantly higher in the case of TFA compared to TRA (1.9% vs. 0.7%; *p* < 0.001), and also in the case of radial left compared to radial right (0.43% vs. 1.05%; *p* < 0.001). Moreover, the rate of pre-hospital cardiac arrest prior to PSM analysis was significantly greater in the group of patients with TFA compared to TRA (1.18% vs. 0.4%; *p* < 0.001), and also in the group of patients with right compared to left TRA (0.52% vs. 0.29%; *p* = 0.02). A comparison between selected clinical indices according to the TRA and TFA after PSM analysis is presented in Table 2, while similar results after inclusion into PSM analysis Killip class grade and cardiac arrest before admission to hospital are presented in Table 3. 

### 3.2. Procedural Indices

Before PSM analysis, the overall amount of contrast dose was significantly higher in the TFA group in comparison to TRA (*p* < 0.001), and the radiation dose was higher in the TRA group (*p* < 001). The thrombectomy rate was also significantly higher in the TFA group when compared to TRA (*p* = 0.002), while the patency of the target artery was significantly higher in the TRA group (*p* < 0.001). This is presented in Table 1. The comparison of selected procedural indices after PSM and inclusion into PSM Killip class grade and the incidence cardiac arrests before admission to the hospital are presented in Table 2 and Table 3. 

The rate of procedure-related complications differed between patients from the TRA and TFA groups before PSM analysis. The death rate was significantly higher in the TFA group when compared to TRA (0.35% vs. 0.16%; *p* < 0.001), as well as cardiac arrest (0.67% vs. 0.29%; *p* < 0.01) and the coronary artery perforation rate (0.27% vs. 0.16%; *p* = 0.03), whereas the rate of periprocedural allergic reactions and coronary artery dissections was higher in the TRA group compared to TFA (0.16% vs. 0.04%; *p* < 0.001 and 0.16% vs. 0.08%; *p* = 0.048). There were no significant differences between the TRA and TFA groups regarding the frequency of myocardial infarctions (0.13% vs. 0.2%; *p* = 0.1), no reflows (0.53% vs. 0.62%; *p* = 0.26) or puncture site bleeding (0.11% vs. 0.16%; *p* = 0.26). There was only one incidence of procedure-related cerebral stroke, which occurred in the TFA group. 

### 3.3. Femoral and Radial Access

**Figure 2:**
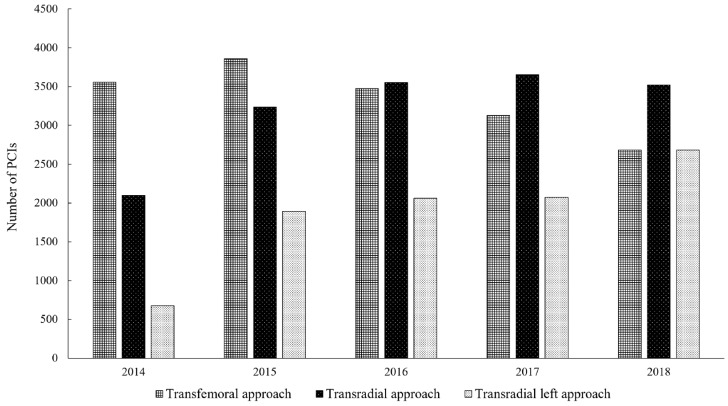
Number of PCIs performed in patients previously treated withCABG according to vascular access in following years (2014-2018). PCI = percutaneous coronary intervention.

**Figure 3:**
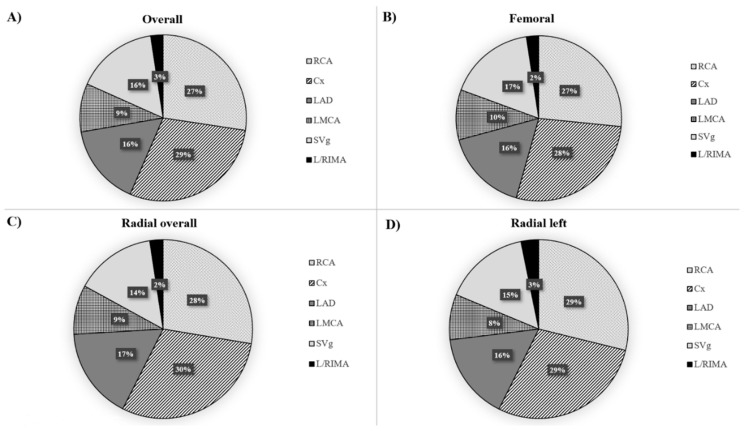
Percentage of PCIs performed on individual vessels depending on the type of vascular access in patients after CABG operation and treated with PCI: (**A**) Overall group; (**B**) Femoral approach; (**C**) Radial approach; (**D**) Left radial approach. Cx = circumflex branch; LAD = left anterior descendent branch; LMCA = left main coronary artery; L/RIMA = left/right internal mammary artery; RCA = right coronary artery; SvG = saphenous vein graft.

The participation of particular vascular accesses in the presented study and its relationship in following years for the patients included in the conducted analysis is presented in Figure 2. Furthermore, the type of vessel treated according to the used vascular access is shown in Figure 3.

Both groups were initially quite balanced, with the highest standardized difference of 10.3% for indication. Propensity scores were obtained using logistic regression taking age, sex, weight, diabetes, history of MI, PCI, smoking, psoriasis and indication into account. After matching, the highest standardized difference was observed for indication (5%). 

**Figure 4:**
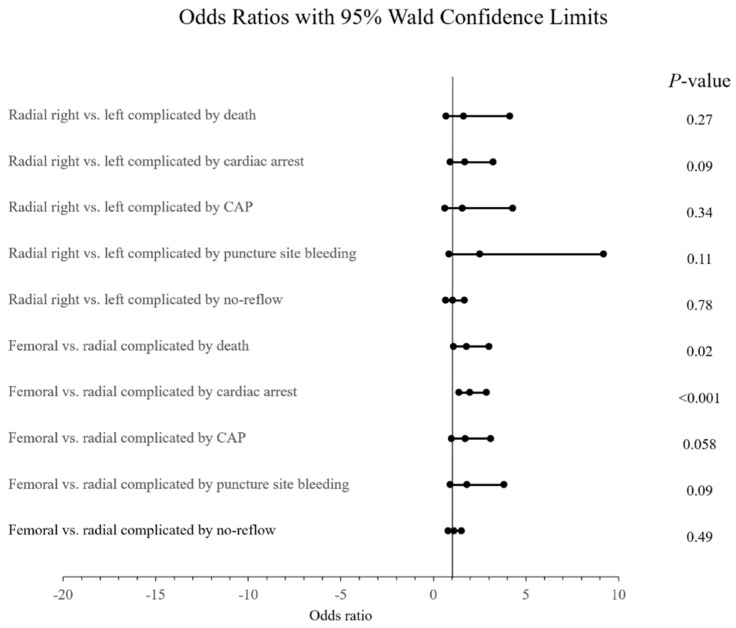
The relationship of femoral vs. radial and radial right vs. radial left vascular access in patients with prior CABG and treated with PCI according to selected procedural related complications. CAP = coronary artery perforation.

The authors have observed that femoral access was associated with 1.8 (95% CI: 1.1 –3; *p* = 0.02) increased odds for death and 1.98 (95% CI: 1.39–2.88; *p* < 0.001) increased odds for cardiac arrest. Such a significant association was not observed for coronary artery perforation (OR: 1.73; 95% CI: 0.99–3.09, *p* = 0.059), puncture site bleeding (OR: 1.83; 95% CI: 0.92–3.82, *p* = 0.09] or no reflow (OR: 1.12; 95% CI: 0.82–1.53, *p* = 0.49). The results were subjected to primary (model A) and sensitivity (models B and C) analyses, the results of which are presented in Figure 4. 

After inclusion into PSM Killip class grade and cardiac arrests which occurred before admission to the hospital, the relationship between procedure-related complications and vascular access was only noticed in the case of procedure-related cardiac arrests (OR: 1.55; 95% CI: 1.07–2.28, *p* = 0.022] and allergic reactions (OR: 0.19; 95% CI: 0.05–0.53, *p* = 0.003], while borderline for coronary artery perforations (OR: 1.82; 95% CI: 1.005–3.43, *p* = 0.053], puncture site bleeding (OR: 2.14; 95% CI: 0.99–4.98, *p* = 0.059) and overall complications rate (OR: 1.22; 95%CI: 0.99–1.49, *p* = 0.053). The relationships were the following for deaths (OR: 1.53; 95% CI: 0.87–2.79, *p* = 0.14), no reflows (OR: 0.92; 95% CI: 0.65–1.29, *p* = 0.63), myocardial infarctions (OR: 1.5; 95% CI: 0.73–3.21, *p* = 0.27) and arterial dissections (OR: 0.62; 9%% CI: 0.24–1.47, *p* = 0.29]. 

### 3.4. Right and Left Radial Access

The comparison of general patient’s characteristics between patients treated from right and left radial access after PSM analysis is presented in Table 4. 

The authors have observed, when comparing right radial access to left, that radial access was associated with 64% increased odds for death, the results varying from a 21% decrease to as high as a 4-fold increase (*p* = 0.27). There was a 70% increase in the odds for cardiac arrest, varying from a 7% decrease to as high as a 3-fold increase (*p* = 0.09). For coronary artery perforation, a 58% increase in odds occurred. This varied from a 38% decrease to as high as a 4-fold increase (*p* = 0.34). There was a 2.5-fold increase in the odds for puncture site bleeding, which varied from a 16% decrease to as high as a 9-fold increase (*p* = 0.12), and a 6% increase in the odds for no reflow, varying from a 32% decrease to a 68% increase (*p* = 0.79). The results were considered in primary (model A) and sensitivity (model B) analyses, the results of which are presented in Figure 4. 

## 4. Discussion

The main finding of the current study is that the higher incidence of periprocedural cardiac arrests can be found in the TFA group in comparison to TRA, even after inclusion into PSM Killip class grade and the occurrence of cardiac arrest before admission to the hospital. No such significance was confirmed for other periprocedural complications, which included deaths and no reflows, except for allergic reactions. While there was borderline significance for the increased rate of coronary artery perforations, the overall complications rate and puncture site bleedings in the TFA group when compared to TRA. None of the assessed periprocedural complications were found to be significantly related to right or left radial access before and after considering features of high-risk patients. The TFA was related to a significantly higher contrast dose, with lower radiation exposure in comparison to the TRA. 

Patients with a history of CABG surgery tend to be older and present a greater comorbidity burden when compared to those undergoing angiography and PCI for native coronary artery disease [11]. It has been demonstrated in several studies that, despite the increasing trend of more frequent radial access use in recent years, in the overall group of patients treated with PCI, femoral access still prevails in the group of patients with prior history of CABG, where only a small percentage of patients have been treated percutaneously with TRA [9]. There is visible reluctance on the part of operators to change habits related to femoral radial access in the group of patients with prior history of CABG. This is often the consequence of unreasonable fears of a higher rate of procedure-related complications and a high crossover rate from the radial to the femoral approach. TFA is associated with a higher incidence of patients in severe conditions, mainly due to the fact that high-risk patients are more often treated with this approach. This is a result of the fact that older operators are attached to long-standing habits regarding the treatment of severe patients, often after cardiac arrest, treated with mechanical ventilation and in a severe general condition. The latter is caused, for example, by a cardiogenic shock, which needs to be treated with vasopressors. Sometimes, operators do not consider the use of radial access, which could be beneficial in some cases. Similarly, in the authors’ work, the frequency of patients in Killip class IV in the initial group of patients prior to PSM was significantly higher in the case of femoral access compared to radial and in the case of left radial compared to the right radial approach. Moreover, the rate of pre-hospital cardiac arrest before PSM analysis was significantly greater in the group of patients with TFA in comparison to TRA and in the group of patients treated with right compared to left TRA. After inclusion of those two indices, there was only a significantly higher rate of procedure-related cardiac arrests in the TFA group when compared to TRA. Such data concerning high-risk patients assessed before treatment with PCI certainly do not go unnoticed. Even after PSM modification, they undoubtedly contribute to a higher residual cardiovascular mortality risk burden in the group of patients with TFA compared to TRA and right in comparison to left TRA. 

Publications concerning the relationship between the type of vascular access and procedural mortality, as well as the cardiac arrest rate, are very limited, and those that are available refer to trials performed on small number of patients, which substantially weakens the resulting conclusions. This is in opposition to TRA and TFA in the general group of patients treated with PCI. However, in this cohort of patients there was still an increase, and it has been demonstrated in previously published studies that during the study period, PCI in patients with prior history of CABG represented 17.5% of the total PCI volume [12]. The PCI target was the native coronary artery in 62.5% and the bypass graft in 37.5%: SVG (34.9%), arterial graft (2.5%), or both arterial graft and SVG (0.2%) [12]. Similar results were obtained in the current study, in which almost 90% of PCIs were proceeded by coronary angiography/bypassography. Compared to patients undergoing native coronary artery PCI, those undergoing bypass graft PCI had higher-risk characteristics and more procedural complications [12]. Several parameters were found to be associated with PCI on bypass grafts and on the native arteries regarding a greater in-hospital mortality rate [12]. This could play a crucial role, especially when considering the fact that this group of patients is at greater cardiovascular risk at baseline [13]. The decrease in mortality among patients treated with PCIs is of special importance due to the fact that patients following CABG demonstrated poorer prognosis after surgery compared to percutaneous revascularization [14]. They also had poorer procedural and postprocedural clinical outcomes [15]. Al Suwaidi et al., with the use of multivariate logistic regression analysis for the adjustment of differences concerning the baseline characteristics, stated that the treatment of vein graft was independently associated with adverse cardiac events, although prior history of CABG itself was not. They concluded that primary PCI for acute myocardial infarction in patients with previous CABG is associated with higher adverse events largely attributable to adverse baseline clinical characteristics and treatment using vein graft [16]. Burzotta et al. demonstrated that the homolateral transradial approach facilitates left internal mammary artery evaluation in patients with history of previous CABG surgery undergoing coronary angiography [17]. The current analysis suggests that the safest vascular access in patients with prior history of CABG and treated with PCI is the right radial approach. However, this finding may seem implausible. Due to the fact that a great number of patients were bypassed with the internal left mammary artery, the left radial approach is found to be more favorable for many reasons: easier cannulation of the left internal mammary artery, lower amount of contrast and shorter fluoroscopy time. 

In some of the published studies, it was revealed that among patients who had previously undergone CABG surgery, TRA coronary angiography was associated with greater contrast use, longer procedure time and greater access crossover and operator radiation exposure compared to TFA angiography [18]. However, in more recently published research including patients undergoing coronary angiography or interventions on vein saphenous grafts, it has been demonstrated that TRA is associated with lower contrast volume at experienced centers in comparison to TFA, and there were no differences in fluoroscopy time between TRA and TFA [13]. This was also visible in the current analysis, in which the contrast amount was lower in the TRA group when compared to TFA. In a study published by Michael et al., TRA was associated with higher radiation exposure, which is similar to findings of previous studies [8,12,18,19]. Michael et al. found that, from an operator perspective, TRA was associated with significantly greater operator radiation exposure during diagnostic angiography when compared with TFA [18]. They concluded that this may be at least partially explained by increased fluoroscopy time required to engage bypass grafts from TRA. Another likely contributing factor is the use of left radial access, which often requires the operator to “bend over” the patient and, hence, be more exposed to radiation [18]. Increased operator radiation exposure with TRA is described in multiple previous studies and is a cause for concern because, over time, it can lead to significant adverse health consequences [18]. The authors of this study also confirmed that radiation exposure was higher in the TRA group compared to TFA. This relationship was confirmed in some of the later published studies involving experienced operators. It was also revealed that despite the decrease in total procedure time for radial cases with the level of training, the total radiation dose did not decrease for coronary angiography in the general population [20]. As a confirmation, in the currently analyzed study, the contrast amount and radiation exposure were significantly higher in the TRA group compared to TFA, as well as in the left TRA when compared to the right before PSM analysis. 

## 5. Limitations

Some study limitations and strengths should be taken into account. Periprocedural complications were reported by the first operators performing PCI. In the presented study, not all complications related to PCI are included, due to the fact that some patients presented those complications after leaving the catheterization laboratory, or even a few days later, until discharge from the hospital. Moreover, the diagnosis of the periprocedural complications remained at the operator’s discretion. These two issues undoubtedly lead to an underestimation of the actual number of procedure-related complications. Additionally, the reporting of the PCI could add some misleading data, due to the fact that in some cases, operators were not able to report data. They were reported by other members of the catheterization laboratory staff, which included technicians, nurses or residents. The inclusion of a number of other risk factors in the analysis related to the occurrence of perioperative cardiac arrest, and not available in the assessed data (left ventricular ejection fraction, severity of renal failure, severe failure of other organs, number of patent arteries, etc.), may significantly change the current results.

One of the most powerful strengths of the current study is the large number of participants, which is rare when considering the analyzed group of patients. Considering this, it may be concluded that the most visible trends remain stable, even in the case of the lack of some data caused by their improper collection.

## 6. Conclusions

In the presented study, it has been confirmed that there is a statistical association between femoral access and a higher incidence of periprocedural cardiac arrests in comparison to radial access, even after inclusion into PSM Killip class grade and cardiac arrests before admission to hospital in the group of patients with prior history of CABG and treated with PCI. Borderline significance was demonstrated for puncture site bleeding, coronary artery perforations and the overall complications rate. There are no significant differences in periprocedural complication rates for particular complications between right and left radial vascular access before and after inclusion into PSM Killip class grade and cardiac arrest rate before admission to hospital. Femoral vascular access is related to a significantly higher contrast dose and significantly lower radiation exposure in comparison to radial access.

## Figures and Tables

**Table 1:** General patient characteristics according to radial and femoral access in the overall group of patients prior to propensity score matching.

Selected Indices	Radial Artery *n* = 17,609	Femoral Artery *n* = 15,154	*p* Value
Age, years	69.8 ± 8.8	69.5 ± 8.5	0.002
Gender, males	13,560 (77)	11,270 (74.4)	<0.001
Weight, kg	81.8 ± 16.5	81.1 ± 16.5	<0.001
Diabetes	5957 (33.8)	4832 (31.9)	<0.001
Prior cerebral stroke	763 (4.3)	646 (4.26)	0.75
Prior myocardial infarction	10,201 (57.9)	8367 (55.2)	<0.001
Prior PCI	10,031 (57)	8876 (58.6)	0.003
Smoking	1971 (11.2)	1549 (10.2)	0.004
Hypertension	13,910 (79)	11,929 (78.7)	0.54
Kidney failure	1719 (9.8)	1513 (10)	0.5
Diagnosis			
stable angina	6324 (36)	4687 (31)	<0.001
unstable angina	6640 (37.8)	6099 (40.3)	<0.001
NSTEMI	3167 (18)	2899 (19.1)	0.008
STEMI	1207 (6.8)	1235 (8.2)	<0.001
others	242 (1.4)	211 (1.4)	0.89
Coronary angiography			
SVD	4937 (33.2)	4644 (35.3)	<0.001
MVD	6945 (46.8)	6328 (48.2)	0.02
LMCA +/− MVD	2968 (20)	2171 (16.5)	<0.001
Imaging studies			
fractional flow reserve	183 (1.04)	103 (0.7)	<0.001
intravascular ultrasound	281 (1.6)	220 (1.4)	0.28
optical coherence tomography	28 (0.1)	23 (0.1)	0.86
Thrombectomy	226 (1.3)	257 (1.7)	0.002
Rotablation	194 (1.1)	164 (1.1)	0.86
Acetylsalicylic acid before PCI	5974 (33.9)	5125 (33.8)	0.83
TIMI 2/3 before PCI	12,655 (74.2)	10,347 (70.5)	<0.001
Contrast, ml	204.7 ± 96.9	213 ± 99.8	<0.001
Radiation dose, mGy	1309.8 ± 1117.2	1289.5 ± 1051	<0.001

LMCA: left-main coronary artery; MVD: multi-vessel coronary artery disease; NSTEMI: non-ST segment elevation myocardial infarction; PCI: percutaneous coronary intervention; STEMI: ST segment elevation myocardial infarction; TIMI: thrombolysis in myocardial infarction.

**Table 2:** General patient characteristics according to radial and femoral access in the overall group of patients following propensity score matching.

Selected Indices	Radial Artery *n* = 12,261	Femoral Artery *n* = 12,261	*p* Value
Age, years	69.7 ± 8.8	69.5 ± 9	0.14
Gender, males	9225 (75.2)	9134 (74.5)	0.18
Weight, kg	81.7 ± 14.4	81.4 ± 14.4	0.1
Diabetes	3984 (32.5)	3933 (21.1)	0.49
Prior cerebral stroke	538 (4.4)	552 (4.5)	0.68
Prior myocardial infarction	6938 (56.6)	6830 (55.7)	0.17
Prior PCI	7059 (57.6)	7078 (57.7)	0.81
Smoking	1281 (10.4)	1289 (10.5)	0.88
Hypertension	9667 (78.8)	9733 (79.4)	0.3
Kidney failure	1175 (9.6)	1257 (10.3)	0.08
Diagnosis			0.006
stable angina	3574 (29.1)	3447 (28.1)	0.07
unstable angina	5270 (43)	5254 (42.9)	0.84
NSTEMI	2434 (19.9)	2432 (19.8)	0.98
STEMI	793 (6.5)	938 (7.7)	<0.001
others	190 (1.5)	190 (1.5)	1
Coronary angiography			<0.001
single-vessel disease	4063 (33.1)	4351 (33.5)	
multi-vessel disease	5745 (46.9)	5882 (48)	
LMCA +/− MVD	2453 (20)	2028 (16.5)	
Imaging studies			
fractional flow reserve	115 (0.9)	83 (0.7)	0.03
intravascular ultrasound	141 (1.1)	131 (1.1)	0.58
optical coherence tomography	22 (0.2)	18 (0.1)	0.63
Thrombectomy	200 (1.6)	236 (1.9)	0.09
Rotablation	79 (0.6)	87 (0.7)	0.58
Acetylsalicylic acid before PCI	4212 (34.4)	4268 (34.8)	0.46
TIMI 2/3 before PCI	8895 (72.5)	8571 (69.9)	<0.001
Contrast, ml	210.6 ± 91.1	218.3 ± 92.8	<0.001
Radiation dose, mGy	1316.5 ± 1107.8	1301.2 ± 1040.6	0.26

LMCA: left-main coronary artery; MVD: multi-vessel coronary artery disease; NSTEMI: non-ST segment elevation myocardial infarction; PCI: percutaneous coronary intervention; STEMI: ST segment elevation myocardial infarction; TIMI: thrombolysis in myocardial infarction.

**Table 3:** General patient characteristics according to radial and femoral access in the overall group of patients following propensity score matching after inclusion in Killip class grade and cardiac arrests at admission.

Selected Indices	Radial Artery *n* = 9661	Femoral Artery *n* = 9661	*p* Value
Age, years	69.7 ± 8.8	69.5 ± 9	0.08
Gender, males	7226 (74.8)	7175 (74.3)	0.41
Weight, kg	81.6 ± 14.1	81.4 ± 14.5	0.36
Diabetes	3187 (33)	3125 (32.3)	0.34
Prior cerebral stroke	419 (4.3)	436 (4.5)	0.57
Prior myocardial infarction	5484 (56.8)	5373 (55.6)	0.11
Prior PCI	5558 (57.5)	5563 (57.6)	0.95
Smoking	1083 (11.2)	1085 (11.2)	0.98
Hypertension	7636 (79.0)	7718 (79.9)	0.14
Kidney failure	1006 (10.4)	1023 (10.6)	0.7
Diagnosis			0.01
stable angina	2483 (25.7)	2369 (24.5)	
unstable angina	4317 (44.7)	4272 (44.2)	
NSTEMI	2039 (21.1)	2075 (21.5)	
STEMI	668 (6.9)	789 (8.2)	
others	154 (1.6)	156 (1.6)	
Cardiac arrest before PCI	59 (0.6)	151 (1.6)	<0.001
Killip class grade before PCI			
1	8810 (91.2)	8658 (89.6)	<0.001
2	667 (6.9)	694 (7.2)	
3	103 (1.1)	125 (1.3)	
4	81 (0.8)	184 (1.9)	
Coronary angiography			0.26
single-vessel disease	3222 (33.4)	3321 (34.4)	
multi-vessel disease	4749 (49.2)	4706 (48.7)	
LMCA +/− MVD	1690 (17.5)	1634 (16.9)	
Imaging studies			
fractional flow reserve	69 (0.7)	60 (0.6)	0.48
intravascular ultrasound	88 (0.9)	84 (0.9)	0.81
optical coherence tomography	18 (0.2)	16 (0.2)	0.86
Thrombectomy	173 (1.8)	195 (2.0)	0.26
Rotablation	41 (0.4)	44 (0.5)	0.82
Acetylsalicylic acid before PCI	3357 (34.7)	3357 (34.7)	1.0
TIMI 2/3 before PCI	6809 (70.5)	6621 (68.5)	0.003
Contrast, ml	216.1 ± 92.3	217.3 ± 91.5	0.35
Radiation dose, mGy	1309.2 ± 1058.9	1299.7 ± 1052.7	0.52

LMCA: left-main coronary artery; MVD: multi-vessel coronary artery disease; NSTEMI: non-ST segment elevation myocardial infarction; PCI: percutaneous coronary intervention; STEMI: ST segment elevation myocardial infarction; TIMI: Thrombolysis In Myocardial Infarction.

**Table 4:** General patient characteristics according to right and left radial access in the overall group of patients following propensity score matching.

Selected Indices	Right Radial Artery *n* = 6420	Left Radial Artery *n* = 6420	*p* Value
Age, years	69.7 ± 8.8	69.9 ± 8.8	0.22
Gender, males	4879 (76)	4912 (76.5)	0.51
Weight, kg	82.1 ± 14.6	82.1 ± 14.3	0.88
Diabetes	2176 (33.9)	2189 (34.1)	0.82
Prior cerebral stroke	299 (4.7)	268 (4.2)	0.2
Prior myocardial infarction	3730 (58.1)	3756 (58.5)	0.65
Prior PCI	3806 (59.3)	3749 (58.4)	0.31
Smoking	729 (11.4)	748 (11.7)	0.61
Hypertension	5083 (79.2)	5109 (79.6)	0.58
Kidney failure	635 (9.9)	634 (9.9)	1.0
Diagnosis			0.67
stable angina	2152 (33.5)	2081 (32.4)	0.18
unstable angina	2618 (40.8)	2693 (41.9)	0.18
NSTEMI	1176 (18.3)	1173 (18.3)	0.96
STEMI	375 (5.8)	371 (5.8)	0.91
others	99 (1.5)	102 (1.6)	0.88
Coronary angiography			<0.001
single vessel disease	2025 (31.5)	2230 (34.7)	
multi-vessel disease	3163 (49.3)	2856 (44.5)	
LMCA +/− MVD	1232 (19.2)	1334 (20.8)	
Imaging studies			
fractional flow reserve	83 (1.3)	49 (0.8)	0.004
intravascular ultrasound	90 (1.4)	63 (1)	0.03
optical coherence tomography	13 (0.2)	9 (0.1)	0.52
Thrombectomy	104 (1.6)	83 (1.3)	0.14
Rotablation	63 (1)	26 (0.4)	<0.001
Acetylsalicylic acid before PCI	2200 (34.3)	2220 (34.6)	0.72
TIMI 2/3 before PCI	4681 (72.9)	4753 (74)	0.15
Contrast, ml	207.5 ± 91.3	212.3 ± 90.7	0.003
Radiation dose, mGy	1325.5 ± 1166.8	1312.3 ± 1054.2	0.5

LMCA: left-main coronary artery; MVD: multi-vessel coronary artery disease; NSTEMI: non-ST segment elevation myocardial infarction; PCI: percutaneous coronary intervention; STEMI: ST segment elevation myocardial infarction; TIMI: thrombolysis in myocardial infarction.
